# Case Report: A case of epithelioid hemangioendothelioma of the right femoral artery misdiagnosed as arterial occlusion

**DOI:** 10.3389/fsurg.2025.1582444

**Published:** 2025-08-14

**Authors:** Zhengqi Shao, Jian Wang, BaiChuan Wang, HongZhi Hu

**Affiliations:** ^1^ Department of Orthopaedics, Union Hospital, Tongji Medical College, Huazhong University of Science and Technology, Wuhan, China; ^2^ Department of Oncology, Liyuan Hospital, Tongji Medical College, Huazhong University of Science and Technology, Wuhan, Hubei, China; ^3^ Department of Vascular Surgery, Union Hospital, Tongji Medical College, Huazhong University of Science and Technology, Wuhan, China

**Keywords:** epithelioid hemangioendothelioma, vein graft, CAMTA1, WWTR1, surgery

## Abstract

Epithelioid hemangioendothelioma (EHE) is a rare and locally aggressive tumour of vascular endothelial origin, with an estimated prevalence of less than 1 in a million. EHE can arise in any part of the body, most commonly in the liver, lungs, and skeleton, while occurrence in the blood vessels of the extremities is rare. This article reports a rare case of primary epithelioid hemangioendothelioma (EHE) of the right femoral artery. The patient was initially misdiagnosed with lower limb arterial occlusion and treated with stenting and other therapies; however, symptoms recurred, and the diagnosis of EHE was confirmed by pathological biopsy. EHE is very rare and accounts for approximately 1% of all vascular tumours. Based on pathological findings— CD31(+), CD34(−), CAMTA(+)–our patient was diagnosed with WWTR1-CAMTA1 fusion EHE.Treatment of EHE is mainly surgical. In our case, the patient underwent resection of the lesion area and the surrounding soft tissue mass, followed by a reconstruction using a left saphenous vein graft.

## 1 Introduction

Epithelioid hemangioendothelioma (EHE) is a rare and locally aggressive tumour of vascular endothelial origin with an estimated prevalence of less than 1 in a million (1, 2). The median age at which EHE occurs is ∼50 years old (3, 4), with a slightly higher prevalence in women than in men (5). The latest publication of the WHO Classification of Soft Tissue Tumours in 2020 states that EHE is a vascular malignant tumour, as is angiosarcoma (6). EHE can be found in any part of the body, most commonly in the liver, lungs, and bones (7). In contrast, EHE in the vessels of the extremities is rare. Fifty per cent of EHE cases are confined to small vessels and rarely occur in large veins or arteries (8). The prognosis for EHE is relatively positive, with an expected cure rate of 70%–80% if complete resection of EHE is achieved surgically and the margins are observed to be negative microscopically (R0 resection) (9). EHE is often misdiagnosed because of its low prevalence and lack of relevant tumour markers. In this case study, we share a case of EHE that was misdiagnosed as lower limb arterial occlusion, and standardised treatment enabled this patient to avoid lower limb amputation.

## 2 Case report

**Figure 1:**
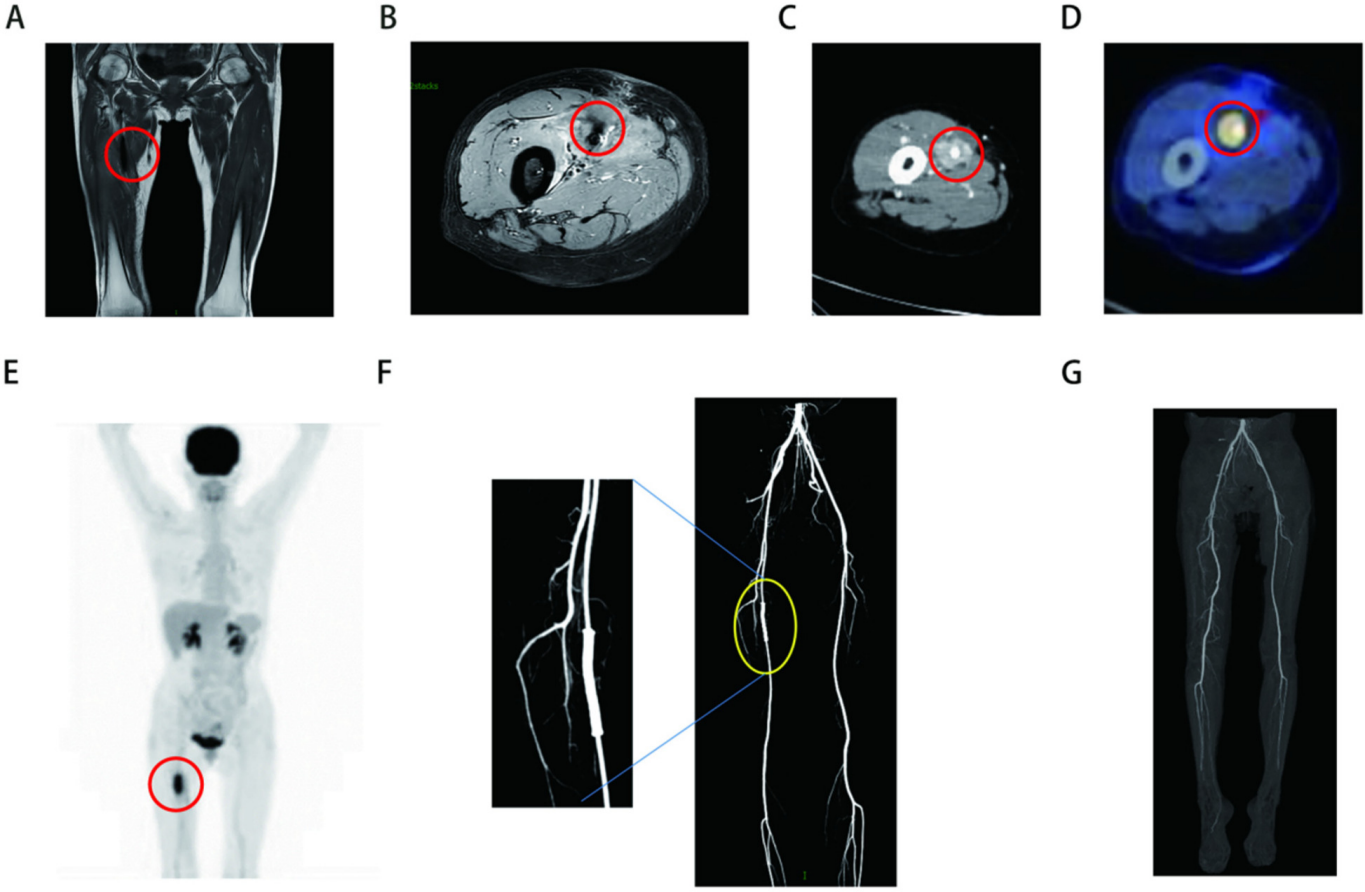
**(A,B)** Preoperative MRI of right thigh (the sequences for **A** and **B** are T1 and T1 fat compression): massive shadow around a stent in the right femoral artery with uneven enhancement. **(C)** Preoperative CT suggests ambiguity around the right femoral artery in the right thigh. **(D,E)** Preoperative PET-CT of the right femoral artery. **(F)** Preoperative CTA of both lower limb arteries suggests changes after stent placement in the middle right femoral artery. **(G)** Postoperative CTA of both lower limb arteries showed postoperative changes in the reconstruction of the right femoral artery with left saphenous vein graft after tumour resection.

**Figure 2:**
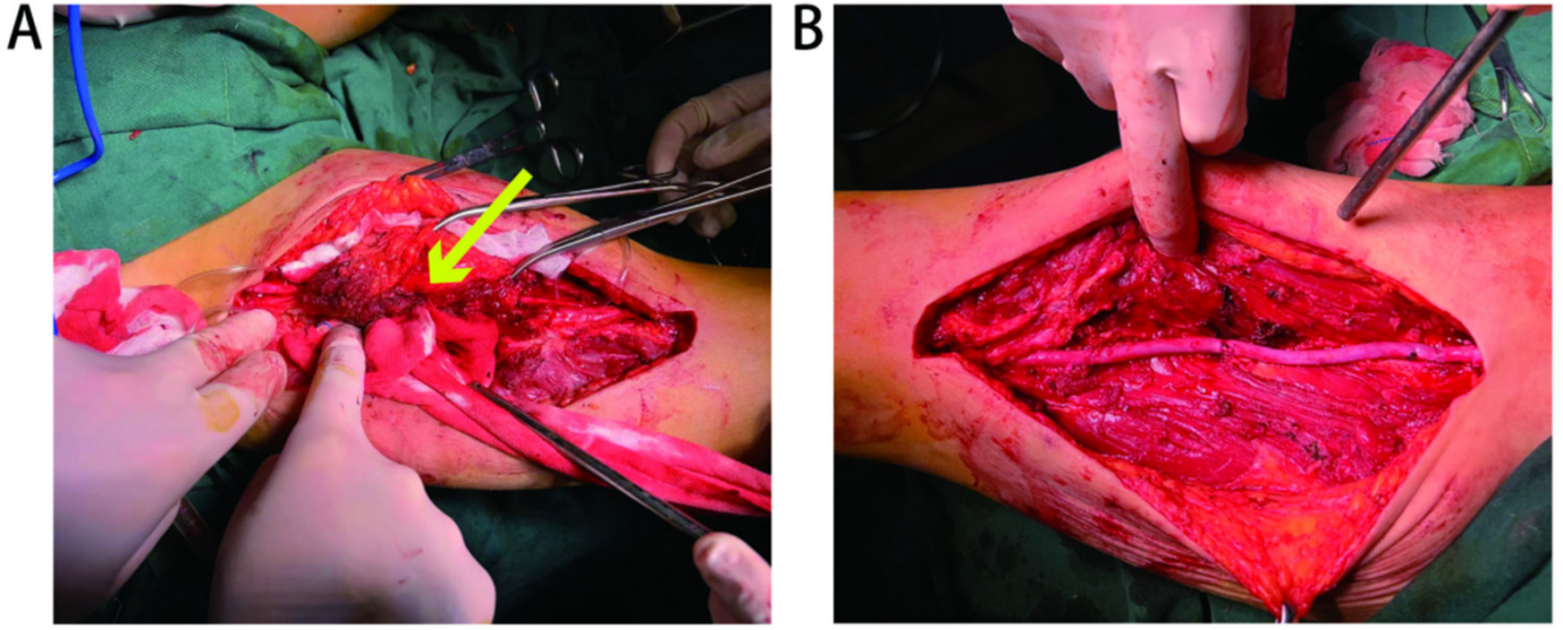
**(A)** Morphology of epithelioid hemangioendothelioma of the right femoral artery is shown by arrows. **(B)** Reconstruction of the right femoral artery with left saphenous vein graft after tumour resection.

**Figure 3:**
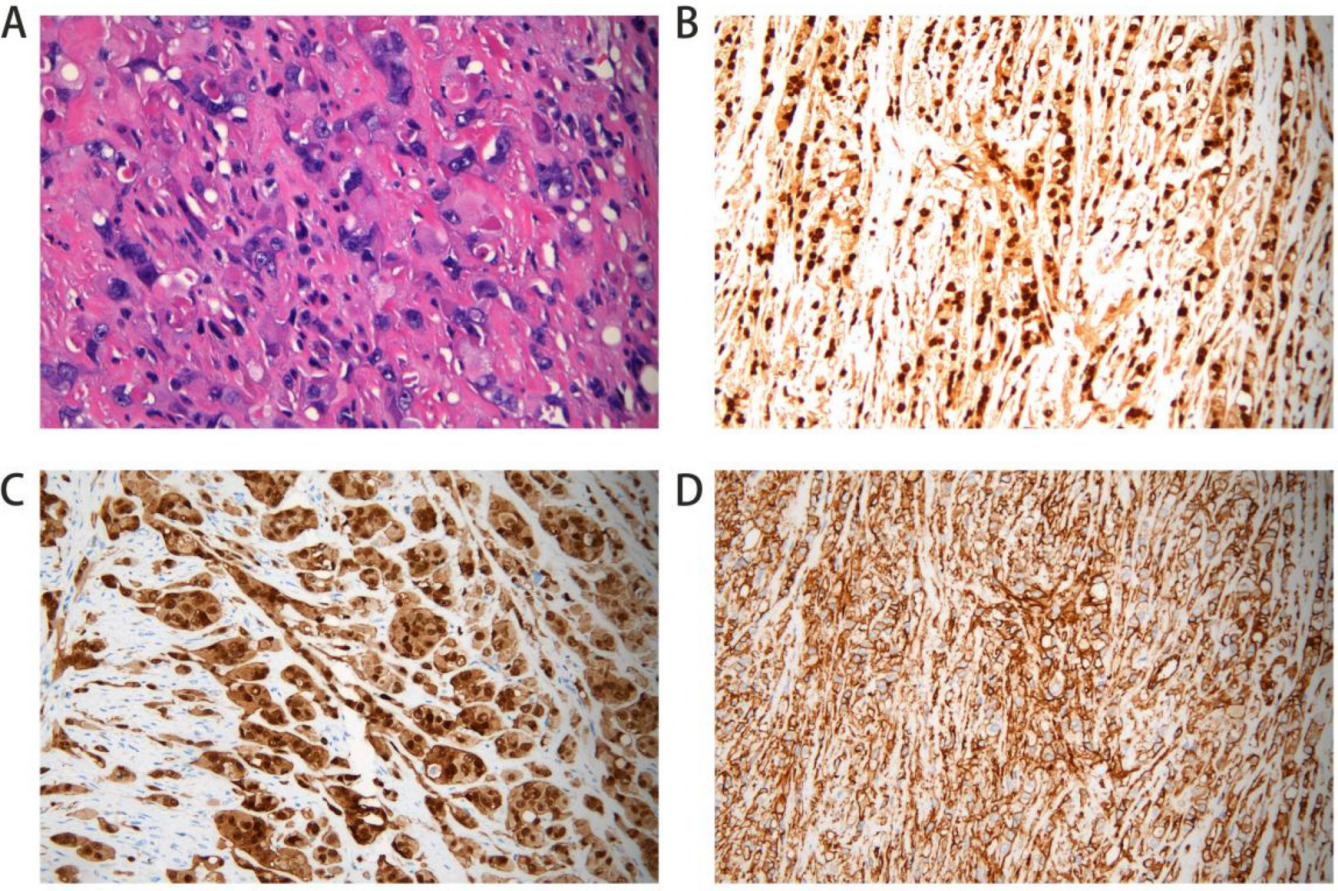
Postoperative pathological section. Hemotoxylin and eosin (HE) staining shows nest-like distribution of tumor cells; cells are epithelial cell-like with increased volume **(A)**. Immunohistochemical stains of the right femoral artery tissue positive for ERG **(B)**. Stain for CD31 was positive **(C)**. CAMTA expression was positive **(D)**.

A 52-year-old woman presented to the Department of Vascular Surgery with right thigh and right lumbar buttock pain for more than 1 year, which had been aggravated for 5 months, and was diagnosed with lower limb arterial occlusion. Lower limb artery balloon dilatation was performed in September 2023, and lower limb artery balloon dilatation angioplasty + stenting was performed in May 2024, after which the pain was relieved for 2 months, and pain and discomfort later developed in the inner and outer right thigh and right lumbar and buttock regions. She was re-evaluated in the Department of Vascular Surgery and was suspected to have a stent-relatd infection. She underwent lower limb arterial exploration in the Department of Vascular Surgery on 27 November 2024. The stent implantation site was blurred with the surrounding tissues, and a specimen of the tissues around the stent was taken for biopsy. Pathology suggested epithelioid hemangioendothelioma of the right thigh. Preoperative MRI of the right thigh suggested a mass shadow around the stent of the right femoral artery with uneven enhancement, considering the possibility of malignant neoplastic lesion (Figures 1A,B). Preoperative CTA of both lower limb arteries revealed atherosclerotic changes in the lower limb arteries and changes after stent placement in the middle section of the right femoral artery (Figure 1F). Before the surgery, we consulted with vascular surgeons and oncologists: no obvious metastases were found, and complete surgical removal of the lesion was recommended. The patient underwent resection of the right femoral artery lesion area and the surrounding soft tissue mass on 23 December 2024, and a saphenous vein graft of equal length was taken from the left medial thigh. The intraoperative morphology of epithelioid hemangioendothelioma of the right femoral artery is shown in Figure 2A. Figure 2B showed the reconstruction of the right femoral artery with left saphenous vein graft after tumour resection. Postoperative CTA of both lower limb arteries was performed: the right thigh lesion was changed after resection + vascular repair, and the right femoral artery showed postoperative changes (Figure 1G). There was a limited effusion and a little pneumatosis around the upper middle segment of the right femoral artery. The right common iliac artery showed slight atherosclerosis. Postoperative pathological findings included epithelioid hemangioendothelioma of the right thigh and no tumour metastasis in the section of the right inguinal lymph node. Immunohistochemical staining showed tumour cells, ERG(+), CD31(+), CAMTA1(+), PCK(−), CD34(−), P53(20%, varying in strength), RB(+), INI-1(+), and Ki67(LI:25%). Immunohistochemical results are shown in Figure 3. A primary right femoral artery epithelioid hemangioendothelioma was identified. During surgery, we completely removed the lesion from the right femoral artery, and the surrounding lymph node biopsy was negative. We recommended regular follow-up visits for observation. Five months after surgery, the patient returned to the hospital for a follow-up visit. She was walking normally, and there were no abnormalities in the movement of her right lower limb. A video of the patient walking after surgery has been submitted in the Supplementary Material. Bilateral lower limb CTA revealed patency of the right femoral artery after great saphenous vein grafting, with no signs of recurrence. Supplementary Figure S1 shows the CTA of both lower limbs at the 5-month follow-up after surgery. Supplementary Figures S1A,B show three-dimensional images of the lower limb vessels, while Supplementary Figures S1C,D show that the right lower limb vessels are patent and no recurrence is observed.

## 3 Discussion

EHE is a subtype of vascular tumour consisting of epithelioid endothelial cells within a distinctive mucohyaline stroma (10). EHE accounts for only 1% of all vascular tumours (2) and shows a moderate female predominance in terms of sex of incidence (4). The incidence of the disease is concentrated in the middle-aged population, while its occurrence in children is extremely rare (11). Regarding the typing of EHE, the majority of EHE cases (>90%) showed WWTR1-CAMTA1 fusions, with CAMTA1 showing diffuse and strong nuclear expression. A small percentage of EHE cases (5%) showed YAP1-TFE3 fusion with diffuse nuclear TFE3 overexpression (12). The etiology of EHE has not been determined, but hepatic epithelioid hemangioendothelioma may be associated with the use of oral contraceptives (13), exposure to vinyl chloride, hepatitis, ascites, and liver injury (14).

The most common presenting symptoms of EHE in the extremities include localised pain and swelling (2). The clinical presentation of our patient was right thigh right lumbar hip pain, which is consistent with the clinical manifestations of EHE. At the same time, the patient's MRI results showed a mass shadow around the stent of the right femoral artery with uneven enhancement. The clinical presentation combined with imaging may remind us of the disease EHE. However, it is worth noting that prior to the first interventional procedure to place the stent, we did not detect any abnormalities around the right femoral artery through bilateral lower extremity CTA. This case of misdiagnosis of lower extremity arterial occlusion provides some lessons learned: overreliance on 3D vascular reconstruction from CTA while neglecting contrast-enhanced axial CT images may lead to misdiagnosis or delayed tumour diagnosis. Histological examination is the gold standard for the diagnosis of this tumour. Biopsy and immunohistochemical tests help in the diagnosis of EHE.EHE shows a typical endothelial phenotype characterised by positivity for CD31, CD34, and von Willebrand factor and occasionally for cytokeratin (15). Although EHE expresses similar molecular markers to angiosarcoma (CD31, CD34, and ERG), angiosarcoma differs greatly from EHE in terms of light microscopy morphology and often exhibits MYC amplification without CAMTA1/TFE3 expression (16). The pathological results of the two times of our patient suggested that CD31(+), CD34(−), and CAMTA(+), combined with the above EHE typing, our patient might be considered as WWTR1-CAMTA1 fusion-type EHE.

The treatment of EHE is primarily surgical, and for limited cancers, surgery is recommended as the first treatment for EHE (17). There are no standardised drug treatment protocols. However, several chemotherapeutic agents have shown good efficacy in the treatment of EHE (14). For patients not suitable for surgical treatment due to poor health, recurrence, or metastasis, VEGF inhibitors [e.g., sorafenib (18), pazopanib (19), or bevacizumab] or other chemotherapeutic agents (including thalidomide, polyethylene glycol liposomal adriamycin, and beat cyclophosphamide) may be used as adjuvant chemotherapy or neoadjuvant chemotherapy. In this case, the patient underwent resection of the right femoral artery in the area of the lesion and the surrounding soft tissue mass, and a saphenous vein graft of equal length was taken from the left medial thigh, as the tumour was relatively confined. Although 30% of this tumour metastasises (half to local lymph nodes and half to the lungs) (20), the prognosis for the disease is relatively good, with an estimated overall mortality rate of less than 20% at 5 years (21, 22). Patients with EHE who undergo complete resection of the lesion and show no evidence of metastasis preoperatively have a good postoperative prognosis. Among the seven cases reported in the literature involving primary involvement of the lower limb arteries or veins (femoral vein or artery), only one case was diagnosed with metastasis (8, 20, 23–27). The patient who experienced metastasis developed delayed pulmonary metastasis 12 years after surgery (pathological examination after resection of the affected lung revealed EHE) (27). The patient in this case underwent a follow-up examination 5 months later and showed satisfactory recovery, with normal walking ability. Bilateral lower limb CTA revealed patency of the right femoral artery after great saphenous vein grafting, with no signs of recurrence. We recommend that the patient continue to undergo bilateral lower limb CTA every 6 months, as well as enhanced CT scans of the lungs and abdomen. Some literature also suggests monitoring through circulating tumour DNA (28).

## 4 Conclusion

This case report describes a rare case of EHE of the right femoral artery. The patient was misdiagnosed with arterial occlusion due to lower limb pain and underwent interventional treatment, but the symptoms recurred. The diagnosis was ultimately confirmed via biopsy. The tumour was completely resected via surgery, followed by autologous saphenous vein grafting for vascular reconstruction (R0 resection). Postoperative pathology revealed CAMTA1 positivity (suggesting the WWTR1-CAMTA1 fusion subtype), with no lymph node metastasis. At the 5-month follow-up, the patient's lower limb function had recovered well, and CTA showed that the graft was patent with no signs of recurrence. This case highlights the need for vigilance in distinguishing EHE from abnormal proliferation after stenting; early radical resection combined with vascular reconstruction can effectively avoid amputation and improve prognosis. Long-term follow-up monitoring of the risk of metastasis is required.

## Data Availability

The original contributions presented in the study are included in the article/Supplementary Material, further inquiries can be directed to the corresponding author/s.
